# Supplementary material for: HAT: Hypergraph analysis toolbox
Source: PLoS Comput Biol. 2023 Jun 5;19(6):e1011190. doi: 10.1371/journal.pcbi.1011190 (PMC10270569; doi:10.1371/journal.pcbi.1011190)
Supplement: S1 File — (PDF) [file pcbi.1011190.s001.pdf]

Supporting Information

Pore-C

Pore-C is a long read sequencing technique designed to capture structural features of genome architecture [1, 2]. It is the most recent extension of chromosome conformation capture (3C) technologies [3]. Pore-C data contains multi-way contacts indicating sets of genomic loci that are colocalized in the nucleus. This reveals insight into the higher-order spatial organization of the genome. The Pore-C assay contains similar processes to previous 3C methods [1]. First, multi-way contacts between any number of genomic loci are ligated in the nucleus. The genomic loci in these regions are detached from their original chromosomes and chained together. The chained regions are sequenced to determine the set of genomic loci that were originally collocated together. Hypergraphs are natural representations of Pore-C data [2]. Individual genomic loci, which can be viewed and binned at any scale for this representation, are represented as vertices in the hypergraph and the colocalization of multiple loci defines a hyperedge. Hi-C data, which captures similar colocalized pairwise relationships, is commonly represented as the adjacency matrix of a graph, but the multi-way contacts of Pore-C necessitate its representation as a hypergraph. The Pore-C assay has already contributed to the field, and new methods of analyzing this data continue to be developed [1, 2].

Hypergraphs

Hypergraph theory extends graph structures to represent multi-way relationships among elements of a set. Mathematically, a graph $\mathcal{G} = \{\mathcal{V}, \mathcal{E}_g\}$ is a set of vertices $\mathcal{V}$ together with a set of edges $\mathcal{E}_g$, where each edge $e \in \mathcal{E}_g$ is a pair of vertices (i.e., $e = (v_i, v_j)$ where $v_i, v_j \in \mathcal{V}$). Graphs are numerically represented as matrices.

Hyperedges model multi-way relationships by allowing hyperedges to contain any number of vertices, expanding beyond the pairwise restrictions of a graph. Formally, a hypergraph $\mathcal{H} = \{\mathcal{V}, \mathcal{E}_h\}$ is a set of vertices together with a set of hyperedges $\mathcal{E}_h$ where each hyperedge $h \in \mathcal{E}_h$ is a subset of vertices (i.e., $h \subseteq \mathcal{V}$). When all hyperedges of a hypergraph have cardinality k, it is referred to as a k-uniform hypergraph. The extension from edges to hyperedges makes hypergraphs more precise representations of data and presents computational advantages.

Numeric Representations of Hypergraphs

The incidence matrix is the primary numerical representation of hypergraphs (Fig 1b). An incidence matrix $\mathbf{H}$ of a hypergraph $\mathcal{H} = \{\mathcal{V}, \mathcal{E}_h\}$ is a $n \times m$ matrix when there are n vertices and m hyperedges. Rows of $\mathbf{H}$ are vertices in the hypergraph, and columns are hyperedges. Each element $\mathbf{H}_{j,i}$ of the incidence matrix is 1 when vertex j is a member of or incident to hyperedge i and 0 otherwise.

A k-uniform hypergraph can also be represented by a tensor (Fig 1c). The adjacency tensor of a hypergraph is the higher-order analogue of a graph adjacency matrix. Mathematically, given a k-uniform hypergraph $\mathcal{H} = \{\mathcal{V}, \mathcal{E}_h\}$ with n vertices, the adjacency tensor is defined as

$$\mathsf{A} \in \mathbb{R}^{\overbrace{n \times \cdots \times n}^{k \text{ times}}} \text{ where } \mathsf{A}_{j_1 \dots j_k} = \begin{cases} \frac{1}{(k-1)!} & \text{if } (j_1, \dots, j_k) \in \mathcal{E}_h \\ 0 & \text{otherwise} \end{cases}.$$

Given the adjacency tensor representation, there are analogue degree and Laplacian tensors based on their pairwise definitions [4, 5, 6].

Hypergraph Expansions

There are two primary pairwise representations of hypergraphs (Fig 1d). Pairwise representations are often helpful to project multi-way interactions into sets of pairwise interactions or to apply standard graph theoretic operations on hypergraphs.

Clique Expansion. The clique expansion algorithm constructs a graph on the same set of vertices as the hypergraph by defining an edge set where every pair of vertices contained within the same edge in the hypergraph have an edge between them in the graph. Given a hypergraph $\mathcal{H} = \{\mathcal{V}, \mathcal{E}_h\}$, then the corresponding clique graph is $\mathcal{C} = \{\mathcal{V}, \mathcal{E}_c\}$ where

$$\mathcal{E}_c = \{(v_i, v_j) |\ \exists\ e \in \mathcal{E}_h \text{ where } v_i, v_j \in e\}.$$

This is called clique expansion because the vertices contained in each $h \in \mathcal{E}_h$ forms a clique in $\mathcal{C}$. While the map from $\mathcal{H}$ to $\mathcal{C}$ is well-defined, the transformation to a clique graph is a lossy process, so the hypergraph structure of $\mathcal{H}$ cannot be uniquely recovered from the clique graph $\mathcal{C}$ alone [6].

Star Expansion. The star expansion of $\mathcal{H} = \{\mathcal{V}, \mathcal{E}_h\}$ constructs a bipartite graph $\mathcal{S} = \{\mathcal{V}_s, \mathcal{E}_s\}$ by introducing a new set of vertices $\mathcal{V}_s = \mathcal{V} \cup \mathcal{E}_h$ where some vertices represent hyperedges. There exists an edge between each vertex $v, e \in \mathcal{V}_s$ when $v \in \mathcal{V}$, $e \in \mathcal{E}_h$, and $v \in e$. Each hyperedge in $\mathcal{E}_h$ induces a star in $\mathcal{S}$. This is a lossless process, so the hypergraph structure of $\mathcal{H}$ is well-defined given a star graph $\mathcal{S}$.

Hypergraph Structural Properties

The following definitions of structural properties of hypergraphs are taken directly from the literature. The average path length [6] of a hypergraph is computed as

$$L_a = \frac{1}{n(n-1)} \sum_{i \neq j} d(v_i, v_j), \tag{S.1}$$

where $d(v_i, v_j)$ is the shortest path between vertices v_i and v_j. For a k-uniform hypergraph, the hypergraph clustering coefficient [4] of vertex v_j is defined as

$$C_j = \frac{|e|e \subseteq \mathcal{N}_j, e \in \mathcal{E}|}{\binom{|\mathcal{N}_j|}{k}}, \tag{S.2}$$

where $\mathcal{N}_j$ is the set of vertices adjacent to v_j. Hypergraph entropy [2, 4] is defined as

$$E(\mathcal{H}) = \sum_{i=1}^{n} -\gamma_i \log(\gamma_i), \tag{S.3}$$

where γ_i are the normalized singular values of the Laplacian tensor of a k-uniform hypergraph or the Laplacian matrix of a hypergraph.

Hypergraph Similarity Measures

Direct hypergraph similarity measures utilize the adjacency tensor to determine the similarity between hypergraphs [6]. The Jaccard and Hamming similarity measures for two k-uniform hypergraphs with n

vertices $\mathcal{H}_1$ and $\mathcal{H}_2$ with adjacency tensors A^1 and A^2, respectively, are defined as

$$\begin{aligned} S_{Jaccard}(\mathcal{H}_1, \mathcal{H}_2) &= 1 - \frac{\sum_{i_1,\dots,i_k} \min(\mathsf{A}^1_{i_1,\dots,i_k}, \mathsf{A}^2_{i_1,\dots,i_k})}{\sum_{i_1,\dots,i_k} \max(\mathsf{A}^1_{i_1,\dots,i_k}, \mathsf{A}^2_{i_1,\dots,i_k})}, \\ S_{Hamming}(\mathcal{H}_1, \mathcal{H}_2) &= \frac{\|\mathsf{A}^1 - \mathsf{A}^2\|}{n^k - n}, \end{aligned} \tag{S.4}$$

where $\|\cdot\|$ denotes the norm of a tensor and $i_1, \dots, i_k$ represent the indices of the elements in the tensors. Feature-based similarity measures compare hypergraphs by exploiting their features or statistics, such as the eigenspectrum of the adjacency tensor and centrality distributions. The centrality- and spectral-based similarity measures are defined as

$$\begin{aligned} S_{Centrality}(\mathcal{H}_1, \mathcal{H}_2) &= \frac{1}{n}\sum_{i=1}^{n} \|c_i^1 - c_i^2\|, \\ S_{Spectral}(\mathcal{H}_1, \mathcal{H}_2) &= \frac{1}{n}\sum_{i=1}^{n-1} \|\gamma_i^1 - \gamma_i^2\|, \end{aligned} \tag{S.5}$$

where c_i^1 and c_i^2 are the centrality scores of the ith vertices in $\mathcal{H}_1$ and $\mathcal{H}_2$, respectively, and γ_i^1 and γ_i^2 are the ordered singular values of the Laplacian tensors associated with $\mathcal{H}_1$ and $\mathcal{H}_2$, respectively.

Hypergraph Controllability

Hypergraph controllability is an extension of graph controllability, which is developed through the use of tensor algebra and polynomial control theory [5]. It is worth noting that hypergraph controllability is only defined for even uniform hypergraphs. Given a k-uniform hypergraph $\mathcal{H}$ with n nodes (k is even), the dynamics of $\mathcal{H}$ with control inputs can be represented by a tensor-based dynamical system

$$\dot{\mathbf{x}} = \mathsf{A}\mathbf{x}^{k-1} + \sum_{j=1}^{m} \mathbf{b}_j u_j, \tag{S.6}$$

where $\mathsf{A} \in \mathbb{R}^{n\times n\times\cdots\times n}$ is the adjacency tensor of $\mathcal{H}$ and $\mathbf{B} = \begin{bmatrix}\mathbf{b}_1 & \mathbf{b}_2 & \dots & \mathbf{b}_m\end{bmatrix} \in \mathbb{R}^{n\times m}$ is the control matrix used to indicate driver nodes. Here the term $\mathsf{A}\mathbf{x}^{k-1}$ refers to the tensor vector multiplications, see detailed definition in [5]. A generalized Kalman's rank condition was derived in order to compute the minimum number of driver nodes required for hypergraph controllability. Let $\mathscr{C}_0$ be the linear span of

$\{\mathbf{b}_1, \mathbf{b}_2, \ldots, \mathbf{b}_m\}$. For each integer $q \geq 1$, define $\mathscr{C}_q$ inductively as the linear span of

$$\mathscr{C}_{q-1} \cup \{\mathsf{A}\mathbf{v}_1\mathbf{v}_2 \ldots \mathbf{v}_{k-1} | \mathbf{v}_l \in \mathscr{C}_{q-1}\}. \tag{S.7}$$

Denote the subspace $\mathscr{C}(\mathsf{A}, \mathbf{B}) = \cup_{q \geq 0} \mathscr{C}_q$. Based on polynomial control theory, the tensor-based dynamical system (S.6) is controllable if and only if $\mathscr{C}(\mathsf{A}, \mathbf{B})$ spans $\mathbb{R}^n$. Detailed algorithms for computing controllability matrices and the minimum number of driver nodes are presented in [5].

References

[1] Deshpande AS, Ulahannan N, Pendleton M, Dai X, Ly L, Behr JM, et al. Identifying synergistic high-order 3D chromatin conformations from genome-scale nanopore concatemer sequencing. Nature Biotechnology. 2022:1-12.

[2] Dotson GA, Chen C, Lindsly S, Cicalo A, Dilworth S, Ryan C, et al. Deciphering multi-way interactions in the human genome. Nature Communications. 2022 Sep;13:5498.

[3] Lieberman-Aiden E, van Berkum NL, Williams L, Imakaev M, Ragoczy T, Telling A, et al. Comprehensive Mapping of Long-Range Interactions Reveals Folding Principles of the Human Genome. Science. 2009 Oct;326(5950):289-93.

[4] Chen C, Rajapakse I. Tensor entropy for uniform hypergraphs. IEEE Transactions on Network Science and Engineering. 2020;7(4):2889-900.

[5] Chen C, Surana A, Bloch AM, Rajapakse I. Controllability of hypergraphs. IEEE Transactions on Network Science and Engineering. 2021;8(2):1646-57.

[6] Surana A, Chen C, Rajapakse I. Hypergraph Similarity Measures. IEEE Transactions on Network Science and Engineering. 2023;10(2):658-74.
