# Supplementary material for: HAT: Hypergraph analysis toolbox
Source: PLoS Comput Biol. 2023 Jun 5;19(6):e1011190. doi: 10.1371/journal.pcbi.1011190 (PMC10270569; doi:10.1371/journal.pcbi.1011190)
Supplement: Attachment — Submitted filename: Hypergraph_Analysis_Toolbox___Reviewer_Response (1).pdf [file pcbi.1011190.s002.pdf]

Response to Reviewer Comments on Manuscript PCOMPBIOL-D-22-01881

Title: HAT: Hypergraph Analysis Toolbox

We thank the editor and the Reviewers for their time and valuable comments, and here we address the issues raised by the reviewers. Please see the following reviewer comments (in black) and our point-by-point responses (in blue). Based on the reviewers' suggestions, we have revised our manuscript significantly, improved the clarity of our work, and addressed errors throughout the text.

Comments by Reviewer #1

In this manuscript, the authors present a novel toolbox to work with hypergraphs, named Hypergraph Analysis Toolbox (HAT). I find the work novel and timely, and I think it can be of potential interest to many scientists working in biology and ecology from the perspective of complex science.

I found the references adequate, and the methodology well presented and explained. The authors have previously developed a code that is publicly available and documented, ready to be used. Given the interest of the subject (the study of many-way interactions in complex networks) and the few tools available, I consider this work worthy of publication in PlosComputational Biology.

I have however some minor corrections and some suggestions that I think can improve the manuscript before it is published.

We thank Reviewer #1 for reviewing our manuscript and thoroughly summarizing our work. Next, we address each of her/his remaining comments in order.

1. L 28: split-pool tagmentation (SPRITE) → Do the authors mean split-pool tag extension? As it is described in the publication where it is presented: SPRITE: a genome-wide method for mapping higher-order 3D interactions in the nucleus using combinatorial split-and-pool barcoding https://www.nature.com/articles/s41596-021-00633-y

   We thank Reviewer #1 for pointing this out. We are referring to the SPRITE assay, and in our group, we sometimes refer to it as split pool tagmentation. We have modified the test to read "Split-Pool Recognition of Interactions by Tag Extension (SPRITE)" for clarity. Additionally, it is our understanding that the reference [1] is the earliest reference to the introduction of the SPRITE assay, but we have included the reference to [2] as well, since it provides a recent discussion of the SPRITE method by the same group that developed it. We have updated the corresponding text in the revised manuscript as follows:

   > *"While classical 3C is pairwise, recent advancements capture multi-way chromatin interactions via proximity ligation (see Pore-C in Supporting Information) [5], Split-Pool Recognition of Interactions by Tag Extension (SPRITE) [6, 7], or multi-contact 3C (MC-3C) [8]."* (Page 2, Line 34-35)

2. L45 to 52: This paragraph could benefit from a little more work. I understand the authors are familiar with how different hypergraph tools were used in the case of the Pore-C data to unveil the higher-order organization of the genome, which is a nice example. However, they just mention other examples from social to ecological networks (references 20 to 27). I think a couple of sentences explaining how these tools were applied in these different systems and what was measured in them could improve the demonstration of the relevance of using hypergraphs in the study of complex systems.

   We thank Reviewer #1 for this valuable suggestion. We have expanded the applications of HAT to include the analysis of neuronal networks within the hypothalamus of a mouse. Additionally, we have provided a concise overview of how HAT can be applied to social networks, biochemical reaction networks, ecological networks, and epidemiological networks. We have updated the corresponding texts in the revised manuscript as follows:

   > *"In addition to examining Pore-C data, HAT was also used to quantify the activity of hypothalamic neurons monitored during a mouse feeding, fasting, and re-feeding experiment [31]. When constructing graph and hypergraph representations of the neuronal network within the hypothalamus for each phase of the experiment, hypergraph entropy proved to be a better indicator of changes in neuronal activity compared to graph entropy [10]. A similar result was also observed from a controllability/observability perspective under the same setting [11, 32]."* (Page 5, Line 107-112)
   >
   > *"Other applications of HAT include detecting influential hubs in social networks [33, 34], gaining insights into the stability and robustness of biochemical reaction networks [35, 36], identifying keystone species in ecological networks [37], and pinpointing control targets in epidemiological networks [38]."* (Page 5, Line 113-115)

3. L56 In reference 34 "Dengyong Zhou, Jiayuan Huang, and Bernhard Schölkopf. Beyond pairwise classification and clustering using hypergraphs. 2005." the name of the journal (or the book editorial etc) is missing.

We thank Reviewer #1 for pointing out this mistake. We have corrected the citation in the revised manuscript as follows:

> *"[28] D. Zhou, J. Huang, and B. Sch ölkopf. Beyond pairwise classification and clustering using hypergraphs. Technical Report 143, Max Planck Institute for Biological Cybernetics, August 2005."* (Page 9, Line 197-198)

4. L57 There is an error in the enumeration, the number 3 is repeated. It now reads "HAT targets the following hypergraph features and problems: 1[..], 2[..], 3[..], 3[..], and 4[..]" (it should be 1,2,3,4,5)

   We thank Reviewer #1 for pointing out this mistake. We have corrected the numbering in the revised manuscript as follows:

   > *"HAT targets the following hypergraph features and problems: (1) construction from data [23, 24, 25], (2) expansion and numeric representation [26, 27, 28], (3) characteristic structural properties (such as entropy [10], centrality [29], distance [12], and clustering coefficients [10]), (4) controllability [11], and (5) similarity measures [12]."* (Page 3, Line 54-57)

5. L60-L66: I encountered a problem, not in the text but running the code of HAT. For what it's worth I'll give a little detail: When trying to obtain the hypergraph of the Karate Club Network, the hyperedges2IM(hyperedges) function is not generating the incidence matrix properly. I fixed the threshold at 0.995 to only obtain 45 hyperedges and then generated the Incidence matrix. However, instead of including 1s and 0s in the proper places it fills the matrix with ones in the upper 35 rows or so, and 0s in the lower 10 rows. I'm using python 3.11, numpy1.23.5 , networkx 2.8.4, and matplotlib 3.7.1.

   We sincerely thank Reviewer #1 for taking the time and effort to verify the correctness of our software. As part of a robust effort to continue the development and maintenance of HAT, we have continued to develop a test suite and debug HAT. Additionally, our documentation page makes an effort to direct users to our GitHub issue page where we able to assist in debugging or identifying and fixing the issues in HAT.

   With respect to this particular issue, we presume the Reviewer is referring to an error found in Tutorial 2, where multi-correlations are used to construct a 3-uniform hypergraph from pairwise data. We believe this particular issue has been fixed in HAT and for this tutorial in the most recent release of HAT. When we run this tutorial on Google CoLab, we have software versions: Numpy 1.22.4, NetworkX 3.0, Matplotlib 3.7.1, and Python 3.9.16. We kindly request the Reviewer to verify if they believe the bug still exist, and if so to report the specific problem to our GitHub issues page so that we are able to better assist in debugging the issue and correcting the software.

6. Characteristic Structural Properties and Similarity Measures: I highly recommend including the equations used to compute these metrics in the supplementary information of this manuscript (or even in a table in the main text). As it is now one needs to go to the original papers where they are presented, and in my case, the institution where I work is not subscribed to some of these journals. It is possible to read them in the arxiv version, but it can be confusing if the authors modified the equation numbering. Hence, in order to improve the clarity of this work, I think it is really important to include the equation of what is being measured together in the same manuscript where the toolkit is presented.

   We thank Reviewer #1 for this critical comment. We recognize the importance of making all our methodologies accessible to the audience of PLOS Computational Biology. We have expanded the supplementary sections by including the associated mathematical equations in Characteristic Structural Properties, Similarity Measures, and Hypergraph Controllability. We have updated the corresponding texts in Supporting Information as follows:

   > *"The following definitions of structural properties of hypergraphs are taken directly from the literature. The average path length [6] of a hypergraph is computed as*
   >
   > $$L_a = \frac{1}{n(n-1)} \sum_{i \neq j} d(v_i, v_j), \tag{S.1}$$
   >
   > *where $d(v_i, v_j)$ is the shortest path between vertices v_i and v_j. For a k-uniform hypergraph, the hypergraph clustering coefficient [4] of vertex v_j is defined as*
   >
   > $$C_j = \frac{|e|e \subseteq \mathcal{N}_j, e \in \mathcal{E}|}{\binom{|\mathcal{N}_j|}{k}}, \tag{S.2}$$
   >
   > *where $\mathcal{N}_j$ is the set of vertices adjacent to v_j. Hypergraph entropy [9,10] is defined as*
   >
   > $$E(\mathcal{H}) = \sum_{i=1}^{n} -\gamma_i \log(\gamma_i), \tag{S.3}$$
   >
   > *where γ_i are the normalized singular values of the Laplacian tensor of a k-uniform hypergraph or the Laplacian matrix of a hypergraph."* (Page 3, Line 51-57)

"Direct hypergraph similarity measures utilize the adjacency tensor to determine the similarity between hypergraphs [6]. The Jaccard and Hamming similarity measures for two k-uniform hypergraphs with n vertices $\mathcal{H}_1$ and $\mathcal{H}_2$ with adjacency tensors A^1 and A^2, respectively, are defined as

$$\begin{aligned} S_{Jaccard}(\mathcal{H}_1,\mathcal{H}_2) &= 1 - \frac{\sum_{i_1,\dots,i_k} \min(\mathsf{A}^1_{i_1,\dots,i_k}, \mathsf{A}^2_{i_1,\dots,i_k})}{\sum_{i_1,\dots,i_k} \max(\mathsf{A}^1_{i_1,\dots,i_k}, \mathsf{A}^2_{i_1,\dots,i_k})}, \\ S_{Hamming}(\mathcal{H}_1,\mathcal{H}_2) &= \frac{\|\mathsf{A}^1 - \mathsf{A}^2\|}{n^k - n}, \end{aligned} \tag{S.4}$$

where $\|\cdot\|$ denotes the norm of a tensor and $i_1,\dots,i_k$ represent the indices of the elements in the tensors. Feature-based similarity measures compare hypergraphs by exploiting their features or statistics, such as the eigenspectrum of the adjacency tensor and centrality distributions. The centrality- and spectral-based similarity measures are defined as

$$\begin{aligned} S_{Centrality}(\mathcal{H}_1,\mathcal{H}_2) &= \frac{1}{n}\sum_{i=1}^{n} \|c_i^1 - c_i^2\|, \\ S_{Spectral}(\mathcal{H}_1,\mathcal{H}_2) &= \frac{1}{n}\sum_{i=1}^{n-1} \|\gamma_i^1 - \gamma_i^2\|, \end{aligned} \tag{S.5}$$

where c_i^1 and c_i^2 are the centrality scores of the ith vertices in $\mathcal{H}_1$ and $\mathcal{H}_2$, respectively, and γ_i^1 and γ_i^2 are the ordered singular values of the Laplacian tensors associated with $\mathcal{H}_1$ and $\mathcal{H}_2$, respectively." (Page 3-4, Line 59-67)

"Hypergraph controllability is an extension of graph controllability, which is developed through the use of tensor algebra and polynomial control theory [5]. It is worth noting that hypergraph controllability is only defined for even uniform hypergraphs. Given a k-uniform hypergraph $\mathcal{H}$ with n nodes (k is even), the dynamics of $\mathcal{H}$ with control inputs can be represented by a tensor-based dynamical system

$$\dot{\mathbf{x}} = \mathsf{A}\mathbf{x}^{k-1} + \sum_{j=1}^{m} \mathbf{b}_j u_j, \tag{S.6}$$

where $\mathsf{A} \in \mathbb{R}^{n\times n\times\cdots\times n}$ is the adjacency tensor of $\mathcal{H}$ and $\mathbf{B} = \begin{bmatrix}\mathbf{b}_1 & \mathbf{b}_2 & \dots & \mathbf{b}_m\end{bmatrix} \in \mathbb{R}^{n\times m}$ is the control matrix used to indicate driver nodes. Here the term $\mathsf{A}\mathbf{x}^{k-1}$ refers to the tensor vector multiplications, see detailed definition in [5]. A generalized Kalman's rank condition was derived in order to compute the minimum number of driver nodes required for hypergraph controllability. Let $\mathscr{C}_0$ be the linear span of $\{\mathbf{b}_1, \mathbf{b}_2, \dots, \mathbf{b}_m\}$. For each integer $q \geq 1$, define $\mathscr{C}_q$ inductively as the linear span of

$$\mathscr{C}_{q-1} \cup \{\mathsf{A}\mathbf{v}_1\mathbf{v}_2\dots\mathbf{v}_{k-1} | \mathbf{v}_l \in \mathscr{C}_{q-1}\}. \tag{S.7}$$

Denote the subspace $\mathscr{C}(\mathsf{A}, \mathbf{B}) = \cup_{q\geq 0}\mathscr{C}_q$. Based on polynomial control theory, the tensor-based dynamical system (S.6) is controllable if and only if $\mathscr{C}(\mathsf{A}, \mathbf{B})$ spans $\mathbb{R}^n$. Detailed algorithms for computing controllability matrices and the minimum number of driver nodes are presented in [5]." (Page 4, Line 69-80)

Finally, we thank Reviewer #1 again for reviewing our manuscript and her/his very insightful and constructive comments, which have helped us significantly improve the quality of our manuscript. We hope our responses above have addressed all her/his concerns in a satisfactory manner.

Comments by Reviewer #2

The software article "HAT: Hypergraph Analysis Toolbox" presents a tool for analysis of multi-way interactions and higher-order structures, addressing hypergraph construction (from experimental data) and respective analysis of structural and dynamic graph properties.

We thank Reviewer #2 for reviewing our manuscript and thoroughly summarizing our work. Next, we address each of her/his remaining comments in order.

1. Although a list of hypergraph libraries is provided, the authors have not mentioned the "HyperG" package in R, that covers a broad variety of tools provided also by HAT. It would be very useful to maybe point to the features that HAT has additional to the already available algorithms.

   We thank Reviewer #2 for bringing HyperG to our attention. A motivation for the development of HAT is to centralize disjoint hypergraph tools across a consistent language since we are not aware at this time of a dominant

hypergraph library in any language, as there is for networks or classical graph libraries. HyperG appears an excellent and well-documented library worth of mention in our article, and we have added the appropriate reference to it in Table 1. As with the other software, there are a number of overlapping features between HyperG and HAT; however, HyperG only utilizes the matrix representation of hypergraphs, unlike HAT which uses the tensor representation as well. Tensor representation makes features such as hypergraph similarity measures and controllability calculations unique to HAT. For instance, even though HyperG and HAT both calculate entropy of a hypergraph, HyperG computes the matrix entropy of the clique expanded graph whereas HAT computes the tensor entropy of the hypergraph. The account of multi-way interactions by HAT is a unique feature relative to HyperG and makes the both useful but substantively different software. Thus, HAT enables increased versatility in representing and analyzing multi-way interactions, offering unique features compared to HyperG.

2. The author note that HAT "contains well-studied and novel mathematical methods for hypergraph analysis" , however from the provided list it is unclear to me which is the novel method that they have developed, or does this refer only to implementing a novel method – I assume the authors refer to controllability? In both cases, giving a mathematical description of the methods implemented would be useful for the users.

   We thank Reviewer #2 for this valuable suggestion. This paper implements methods based on recent literature. We have emphasized this point in the conclusion of the revised manuscript as follows:

   > *"The computational methods provided in HAT include hypergraph controllability and similarity measures from a tensor-based perspective. Additionally, the inclusion of tensor-based hypergraph structural properties (i.e., entropy and centrality), the association of multi-correlations with hypergraphs, and the integration of previously implemented graph expansion and visualization techniques within one software is an advancement over previously disjoint implementations." (Page 5, Line 117-122)*

   With respect to providing a mathematical description, we have modified the writing in Section 3 and expanded the supplementary sections by including the associated mathematical equations of the methods. We have updated the corresponding texts in the revised manuscript as follows:

   > *"The following structural properties of hypergraphs are computed: average distance between vertices is computed based on [12] (see Hypergraph Structural Properties in Supporting Information, Equation S.1); the clustering coefficient is calculated based on [10] (Equation S.2); hypergraph centrality is measured according to methods in [29, 30], which employ a variety of techniques to solve the nonlinear eigenvalue problem. For a uniform hypergraph, entropy is computed according to [10], which defined hypergraph entropy based on the higher-order singular values of the Laplacian tensor (Equation S.3)."* (Page 4, Line 75-81)
   >
   > *"Hypergraph controllability refers to the ability to steer the underlying system of a hypergraph to a desired state by manipulating a subset of nodes (often referred to as driver nodes) [11]. For a uniform hypergraph, the minimum number of driver nodes required for controllability can be computed using the generalized Kalman's rank condition (see Hypergraph Controllability in Supporting Information, Equation S.7)."* (Page 4, Line 83-87)
   >
   > *"A series of structural and feature-based hypergraph similarity measures, including the Hamming Distance, the Jaccard Index, spectral measures, and centrality measures are provided (see Hypergraph Similarity Measures in Supporting Information, Equations S.4 and S.5)."* (Page 4, Line 91-93)

   Please also refer to our response to Comment 6 from Reviewer #1 for the inclusion of mathematical descriptions in Supporting Information.

3. The authors have also chosen to demonstrate the method on a single experimental data set – if that is the case, then it would be useful to assess the advantage of the provided software over existing ones by demonstrating to the readers also with the respective analysis of the data set, and maybe suggesting possible biological implications that can result from analyzing the structural or dynamical properties of the constructed hypergraph.

   We thank reviewer #2 for this critical comment. In Section 2, we have included an additional example of the utility of hypergraphs for studying neuronal activity. Additionally, we have discussed the utility of hypergraphs for both 3C and neural activity to contrast the biological understandings of the systems obtained from a HAT versus other existing software. HAT is able to successfully identify several novel biological results, while currently available graph/hypergraph software fail to identify such biological trends. We have updated the corresponding texts in the revised manuscript as follows:

   > *"In terms of biological implications, hypergraph entropy of chromosome structure has identified bifurcation points that determined cell fate over the course of a cell reprogramming experiment, which remained unidentified with a graph-theoretic approach [10]."* (Page 4-5, Line 102-104)
   >
   > *"When constructing graph and hypergraph representations of the neuronal network within the hypothalamus for each phase of the experiment, hypergraph entropy proved to be a better indicator of changes in neuronal activity compared to graph entropy [10]."* (Page 5, Line 108-111)

Finally, we thank Reviewer #2 again for reviewing our manuscript and her/his very insightful and constructive comments, which have helped us significantly improve the quality of our manuscript. We hope our responses above have addressed all her/his concerns in a satisfactory manner.

References

[1] Sofia A Quinodoz, Noah Ollikainen, Barbara Tabak, Ali Palla, Jan Marten Schmidt, Elizabeth Detmar, Mason M Lai, Alexander A Shishkin, Prashant Bhat, Yodai Takei, et al. Higher-order inter-chromosomal hubs shape 3d genome organization in the nucleus. *Cell*, 174(3):744–757, 2018.

[2] Sofia A Quinodoz, Prashant Bhat, Peter Chovanec, Joanna W Jachowicz, Noah Ollikainen, Elizabeth Detmar, Elizabeth Soehalim, and Mitchell Guttman. Sprite: a genome-wide method for mapping higher-order 3d interactions in the nucleus using combinatorial split-and-pool barcoding. *Nature protocols*, 17(1):36–75, 2022.
